# Numerical characterization of the electronic and optical properties of plumbene/hBN heterobilayer using first-principles study

**DOI:** 10.1039/d2na00918h

**Published:** 2023-05-24

**Authors:** Nishat Tasnim Hiramony, Tanshia Tahreen Tanisha, Sumaiya Jahan Tabassum, Samia Subrina

**Affiliations:** a Department of Electrical and Electronic Engineering, Bangladesh University of Engineering and Technology Dhaka 1205 Bangladesh samiasubrina@eee.buet.ac.bd ssubr002@ucr.edu +880-02-9668054 +880-19-3795-9083

## Abstract

We present a novel plumbene/hexagonal boron nitride (hBN) heterobilayer with intriguing structural, electronic, and optical properties. Three different stacking patterns of the bilayer are proposed and studied under the framework of density functional theory using first-principles calculations. All the stacking configurations display direct band gaps ranging from 0.399 eV to 0.432 eV in the presence of spin orbit coupling (SOC), whereas pristine plumbene possesses an indirect band gap considering SOC. Based on binding energy calculations, the structures are found to be stable and, consequently, feasible for physical implementation. All three structures exhibit low effective mass, ∼0.20*m*_0_ for both electrons and holes, which suggests improved transport characteristics of the plumbene/hBN based electronic devices. The projected density of states reveals that the valence and conduction band peaks around Fermi energy are dominated by the contributions from the plumbene layer of the heterobilayer. Therefore, the hBN layer is a viable candidate as a substrate for plumbene since charge carriers will only travel through the plumbene layer. Biaxial strain is employed to explore the dependence of the electronic properties like bandgap and effective mass of the heterobilayer on applied strain. We find that applied biaxial compressive strain can induce switching from the semiconducting to metallic state of the material. In addition, we explore various optical characteristics of both pristine plumbene and plumbene/hBN. The optical properties of the heterobilayer signify its potential applications in solar cells as well as in UV photodetectors.

## Abstract 2

A novel plumbene/hBN heterostructure exhibits a tunable direct bandgap and low effective mass, whereas plumbene has an indirect bandgap. The intriguing characteristics of the heterostructure have inspired the emergence of novel Pb-based nanoelectronic devices.

## Graphical abstract

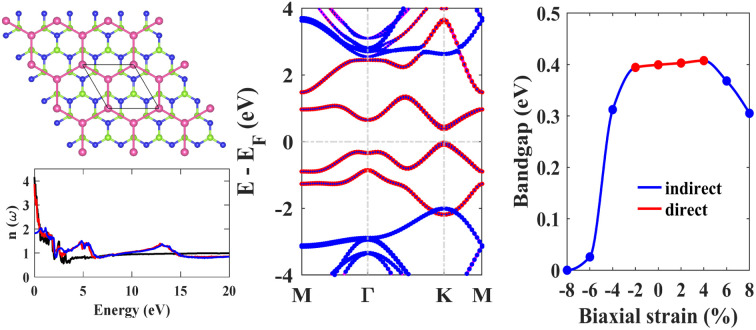

## Introduction

The exceptional properties of graphene prompted researchers to investigate other two-dimensional (2D) materials for similar properties with diverse applications.^1,2^ Group IV 2D materials, such as graphene, silicene, germanene, stanene, and plumbene, have unique electronic, mechanical, and optical properties. Plumbene is graphene's newest cousin, having a buckled honeycomb structure. SOC of 2D materials depends on buckling height.^3^ Among group IV 2D materials, plumbene has the highest buckling height (0.98 Å)^4^ and hence the greatest spin orbit coupling. It is a metal or zero band gap semiconductor when SOC is not considered. However, in the presence of SOC, it shows an indirect bandgap of ∼0.302 eV.^5^ Electron doping^6^ or chemical decoration with different functional groups^7–9^ opens a large bandgap in plumbene and makes it a quantum spin Hall insulator. Decorated plumbene is one of the most attractive choices for quantum spin Hall insulators due to its large SOC. The experimental realization of plumbene^10^ serves as a motivation for exploring novel materials and devices utilizing plumbene and plumbene based heterostructures. Plumbene can be stacked with other monolayers to form heterostructures, developing novel materials with superior properties for futuristic electronic, optical, and spintronic devices. Integrating 2D monolayers with a large band gap material, such as hBN, Al_2_O_3_, or AlN, is reportedly the best method to shield them from ambient conditions.^11^ Hexagonal boron nitride is a preferred substrate due to its atomically flat surface, which is comparatively devoid of dangling bonds.^12^ The low defect density of hBN significantly contributes to improving the device performance.^13^ Furthermore, hBN has been widely used for stacking with other 2D materials and crafting composite materials with highly desirable properties. When a graphene and hBN heterostructure is constructed, the complex has distinct electrical characteristics, such as a small bandgap at the Dirac point, allowing for a wide range of applications in nanoscale devices.^14^ A graphene/hBN in-plane heterostructure has been successfully synthesized on Cu–Ni alloy.^15^ Like graphene/hBN heterostructures, silicene/GaSe,^16^ silicene/hBN,^17^ germanene/hBN,^18^ Ge/2D-SiC,^19^ Ge/BeO,^20^ stanene/hBN,^21^ and stanene/MoS_2_ (ref. 22) heterostructures have also been reported in the literature with their intriguing characteristics. In silicene/hBN, silicene favors electron transport, whereas hBN favors thermal transport making the heterostructure suitable for thermoelectric applications.^17^ In stanene/hBN, electrons transport through the stanene layer making hBN a good substrate for the composite structure.^21^ This motivated us to investigate the heterostructure combining plumbene and hBN. In this work, we first study the structural and electronic properties of the plumbene/hBN heterostructure using first-principles calculations. To find structural properties, three different types of stacking are proposed and the corresponding cells are optimized using appropriate parameters and thresholds. Based on the binding energies of these three structures, the most stable structure is chosen. The bonding mechanism in this structure can be interpreted from the partial density of states and the differential charge densities. Parabolic approximation is used to calculate effective mass from the band structure. These findings are then used to compare how the properties of the structure change after biaxial strain is introduced. It is observed that the bandgap of the heterostructure can be tuned by varying interlayer distances and by applying strain. Then we investigate the optical properties within the context of density functional theory. The computed frequency dependent dielectric functions of the monolayer plumbene and plumbene/hBN heterobilayer are used to compare the optical characteristics of these two structures. These dielectric functions are further used to calculate the optical absorption coefficients and other optical properties of the monolayer and bilayer systems. We also perform classical molecular dynamics simulations to study the thermal stability of the heterobilayer.

## Computational details

All the calculations in this study were carried out using first-principles calculations within the framework of density functional theory. We used Quantum ESPRESSO (QE) to extract the structural, electronic, and optical properties of the proposed heterostructure. The generalized gradient approximation (GGA) within the Perdew–Burke–Ernzerhof (PBE) functional^23^ was adopted to account for the exchange–correlation interaction. Projector Augmented Wave (PAW) pseudopotentials belonging to PSlibrary^24^ were used for geometry optimization and all other calculations in this work. The structures were relaxed until the total energy had reduced to less than 7.3 × 10^−8^ Ry, and the total force reached a value of less than 3.9 × 10^−4^ Ry Bohr^−1^. The relaxation of the cell along the *c*-direction was restricted since the materials are all two-dimensional. The out-of-plane lattice parameter *c* contains a vacuum length of 30 Å to minimize artificial interactions among neighboring periodic images of the structures. A value of 60 Ry was chosen as the kinetic energy cutoff for wavefunction, and 600 Ry was selected as the kinetic energy cutoff for charge density and potential. A 12 × 12 × 1 Monkhorst–Pack grid was implemented here to sample the first Brillouin zone (BZ).^25^ The Broyden–Fletcher–Goldfarb–Shanno^26^ (BFGS) algorithm was used for geometry optimization. van der Waals (vdW) interaction was included in the calculation by enabling Grimme's DFT-D2 classical dispersion force field method in QE. The binding energies of the three stacking configurations considered in this work are determined in order to examine the stability of the structures. A negative value of binding energy signifies the structural stability of the materials. The binding energy is given by,*E*_b_ = *E*_plumbene/hBN_ − *E*_hBN_ − *E*_plumbene_where *E*_b_ is the binding energy per unit cell of the composite structure. *E*_plumbene_, *E*_hBN_, and *E*_plumbene/hBN_ are energies per unit cell for the plumbene monolayer, hBN monolayer, and plumbene/hBN heterostructure, respectively. We also determined the charge density difference, which is defined as,Δ*ρ* = *ρ*_plumbene/hBN_ − *ρ*_hBN_ − *ρ*_plumbene_where *ρ*_plumbene_, *ρ*_hBN_, and *ρ*_plumbene/hBN_ are the charge densities of monolayer plumbene, monolayer hBN, and plumbene/hBN heterostructure, respectively. The effective mass is calculated since it is an essential parameter for characterizing carrier transport within a material. Effective mass is calculated using the following formula,
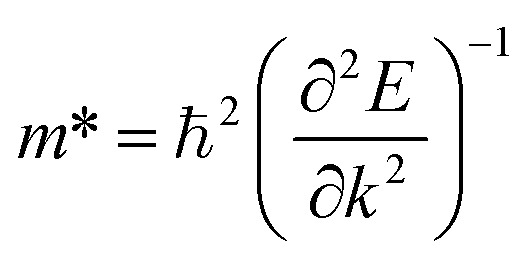


A 36 × 36 × 1 Monkhorst–Pack grid was used in optical calculations to sample the first Brillouin zone. The complex dielectric function *ε*_*α*,*β*_(*ω*) is calculated within the framework of random phase approximation using QE code. *ε*_*α*,*β*_(*ω*) is defined as below^27^:
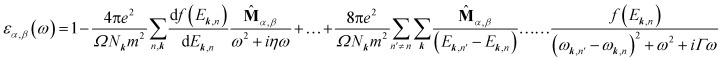
where the double-indexed matrix elements **M̂**_α,β_ contain the result of the momentum operators' evaluation between single-particle Bloch functions obtained by DFT calculation. *E*_***k***,*n*_ and *E*_***k***,*n*′_ denote the energies corresponding to the *k*th point in the valence band *n* and conduction band *n*′, respectively. *Γ* is intersmear, and *η* is intrasmear. *N*_***k***_ is the number of points to sample BZ, and *Ω* represents the volume of the unit cell. All the optical properties are calculated utilizing the complex dielectric function. From the real part (*ε*_real_) and imaginary part (*ε*_img_) of the complex dielectric function, the complex refractive index *N*(*ω*) can be calculated, which is given by,*N*(*ω*) = *n*(*ω*) + *ik*(*ω*)where *n*(*ω*) and *k*(*ω*) are, respectively, the real and imaginary parts of the complex refractive index which are found using the following equations:^28^
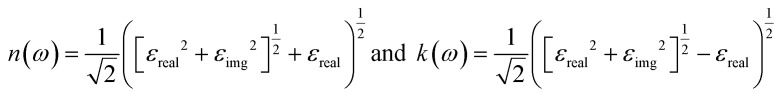


The reflection coefficient for normal incidence of EM waves on a plane surface can be obtained from *n*(*ω*) and *k*(*ω*) by,^28^
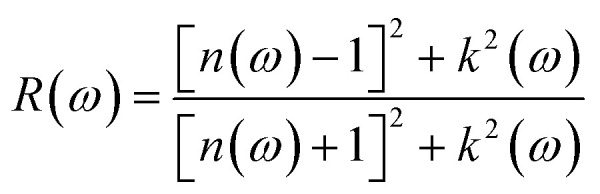


The absorption coefficient can be found from the imaginary part of the refractive index,^28^
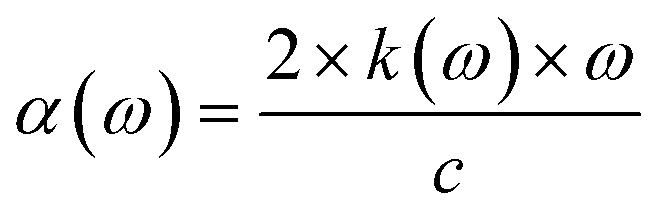


Classical molecular dynamics simulations using empirical force field methods have been performed to investigate the thermal stability of the heterostructure at three different temperatures, which are 300 K, 500 K and 1000 K. A time-step of 1 fs has been used and a total simulation time of 50 ps was selected.

## Results and discussion

The geometry optimized lattice parameter of hexagonal boron nitride is found to be 2.515 Å, and that of monolayer plumbene is found to be 4.863 Å, which are consistent with the reported values of 2.504 Å (ref. 21) and 4.856 Å (ref. 5) respectively. The calculated Pb–Pb bond length is 2.971 Å, and the buckling height of pristine plumbene is 0.972 Å, which are comparable to the reported values of 2.972 Å (ref. 39) and 0.98 Å,^39^ respectively. The obtained lattice parameter of plumbene is 48.3% greater than that of hBN. Thus, to minimize lattice mismatch, plumbene with 1 × 1 lateral periodicity and hBN with 2 × 2 lateral periodicity are taken to compose the plumbene/hBN heterostructure. We carried out variable cell relaxation of the 2 × 2 hBN supercell. The optimized lattice parameter of this supercell found thereafter is 5.025 Å. The lattice mismatch of the heterostructure constructed using this supercell, together with a unit cell of pristine plumbene, is ∼3%, making the structure commensurate. Then we stacked the plumbene unit cell with the hBN supercell and relaxed the structure keeping the cell parameters and the atomic positions of B and N fixed. Therefore, only the epitaxial plumbene monolayer in our commensurate structure became subject to strain as an outcome of the geometry optimization.

**Fig. 1:**
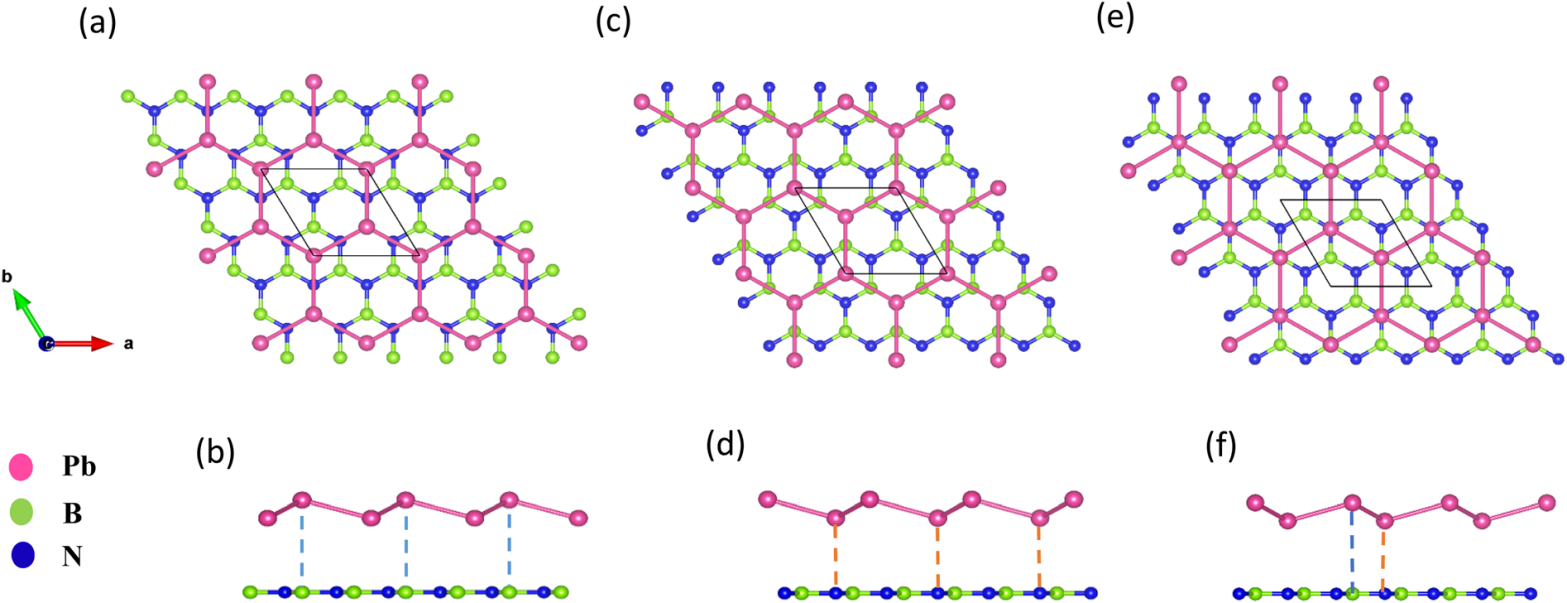
(a) Top view and (b) side view of structure I. (c) Top view and (d) side view of structure II. (e) Top view and (f) side view of structure III.

**Fig. 2:**
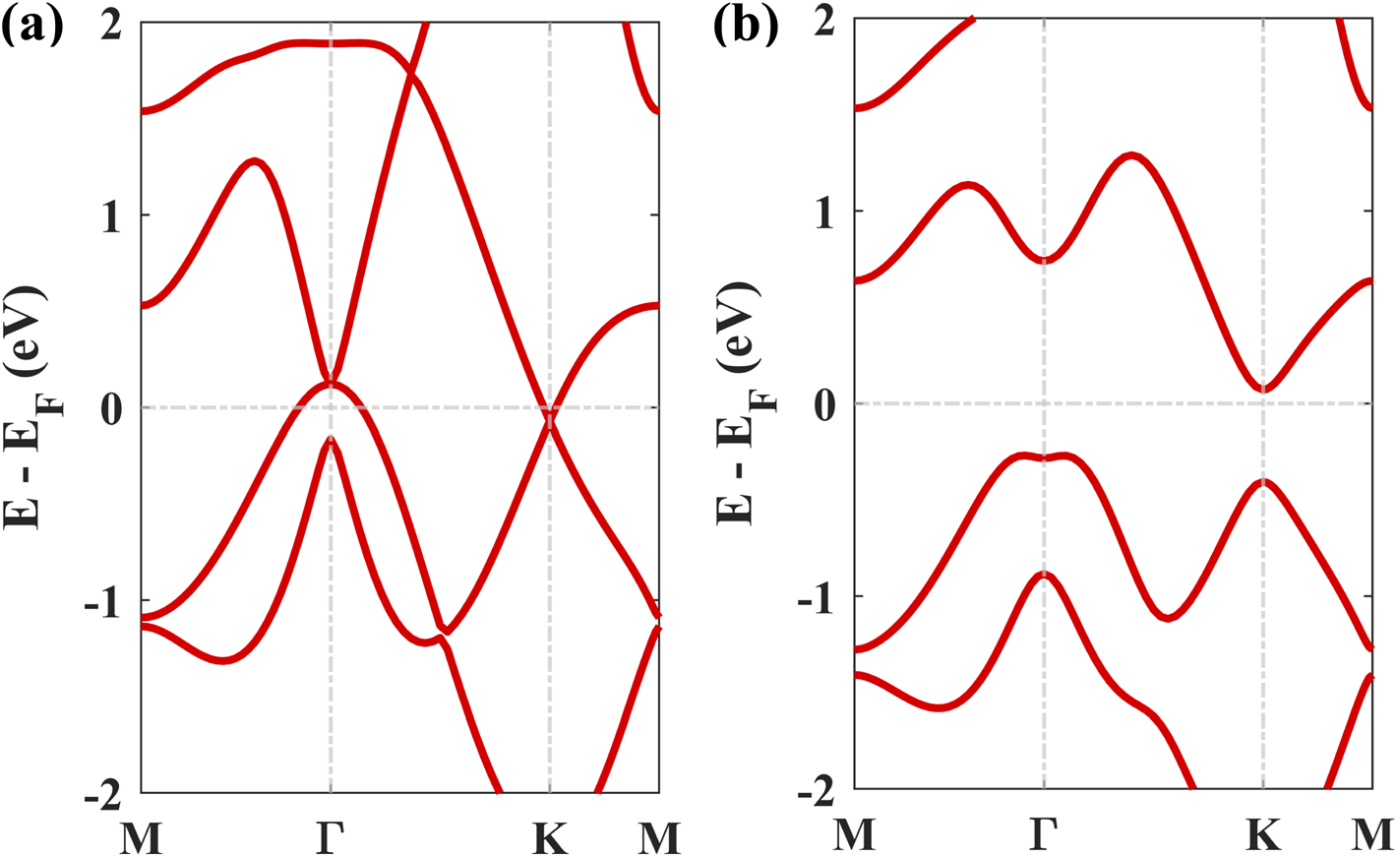
The electronic band structure of plumbene (a) without SOC and (b) with SOC.

**Fig. 3:**
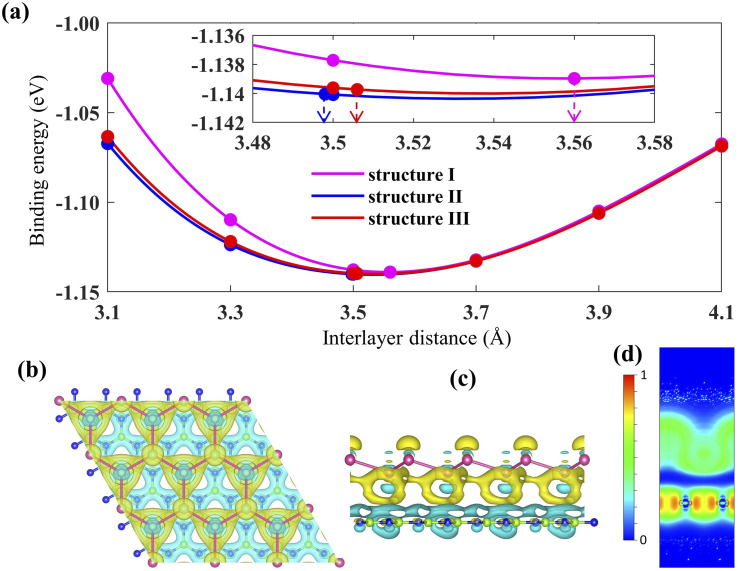
(a) Binding energy per unit cell for the three Pb/hBN configurations with changes in interlayer distance. (b) Top view and (c) side view of charge density difference (isosurface value is 0.00013 e Bohr^−3^; the cyan color denotes charge depletion, and yellow represents charge accumulation). (d) 2D plot of electron localization function (a portion of the unit cell has been omitted vertically for better visualization).

Fig. 1 displays the three kinds of stacking patterns that we have considered in this study: structure I in (a) and (b), structure II in (c) and (d), and structure III in (e) and (f). In structure I, the top Pb atom is positioned over the B atom, and the bottom Pb atom is positioned over the center of the BN hexagon. In structure II, the top Pb atom is placed over the center of the BN hexagon, and the bottom Pb atom is placed over the N atom. In structure III, the top and bottom Pb atoms are positioned over B and N, respectively. Analogous stacking patterns are reported for graphene/hBN,^29^ graphene/germanene,^30^ graphene/stanene, and stanene/hBN^21^ heterostructures. The band structure of the optimized plumbene monolayer is displayed in Fig. 2. In the absence of spin orbit coupling, pristine plumbene has no bandgap. The band structure features a linear dispersion relation around the Dirac cone at *K* point. When SOC is taken into account (Fig. 2(b)), this linear dispersion relation gets destroyed. Besides, the conduction band minimum (CBM) and the valence band maximum (VBM) lie at different *k*-points, implying that monolayer plumbene is an indirect bandgap material. The measured bandgap of plumbene is 0.34 eV, which is close to the reported value of 0.302 eV.^5^ The calculated electron and hole effective masses of plumbene are 0.2255*m*_o_ and 0.4938*m*_o_, respectively. The electron effective mass of the plumbene monolayer is less than that of bulk silicon, which is one of the important parameters for determining electron transport. This indicates that plumbene has the potential for more favorable electron transport as compared to Si. The binding energies of the three configurations are calculated and plotted with respect to interlayer distance in Fig. 3(a). Structures I, II, and III have optimized interlayer distances of 3.560 Å, 3.498 Å, and 3.506 Å, respectively. These optimal interlayer distances are all fairly greater than the Pb–B bond length (2.122 Å)^31^ and the Pb–N bond length (2.75 Å).^32^ Therefore, Pb atoms of the plumbene layer are expected not to form covalent bonds with B and N atoms of the hBN layer. The binding energies of the three patterns are negative, implying the stability of these structures. Structure II has the lowest binding energy, making this structure the most stable one among the three structures. Structures I, II, and III have binding energies of −1.139 eV, −1.14 eV, and −1.1397 eV per unit cell or −569.5 meV, −570 meV, and −569.85 meV per Pb atom, respectively. The binding energies per Pb atom at the optimized interlayer distance are higher than the typical binding energies due to weak vdW interactions.^33^ This indicates that plumbene and hexagonal boron nitride are bound by other interactions, such as electrostatic interaction along with vdW interaction.^34^ Thus, the interfaces between single-layer plumbene and hexagonal boron nitride are energetically stable and easy to implement experimentally.

Next, the charge density difference of plumbene/hBN is plotted in Fig. 3(b) and (c). Because of the buckling height of plumbene, the electric potential energies of the top and bottom Pb atoms differ. The figure reveals that charge depletion occurs near the hBN monolayer, and charge buildup takes place near the plumbene monolayer in the interspace. This implies that charge redistribution is mostly caused by electrostatic repulsion.^35^ As a result, it generates an inherent electric field between the interlayers, which is directed from the hBN toward the plumbene monolayer. This electric field opens a bandgap near the Dirac point in the plumbene/hBN heterostructure. The electron localization function (ELF) is calculated and plotted in Fig. 3(d) to examine the nature of chemical bonds in the heterostructure. The ELF slice with (100) Miller indices has been chosen for plotting the function. ELF takes a value between 0 and 1. Fig. 3(d) clearly shows that ELF has non-zero values in the plumbene and hBN layer, whereas it is zero in the region between the two layers, confirming the absence of any chemical bonding between plumbene and hBN layers.

**Fig. 4:**
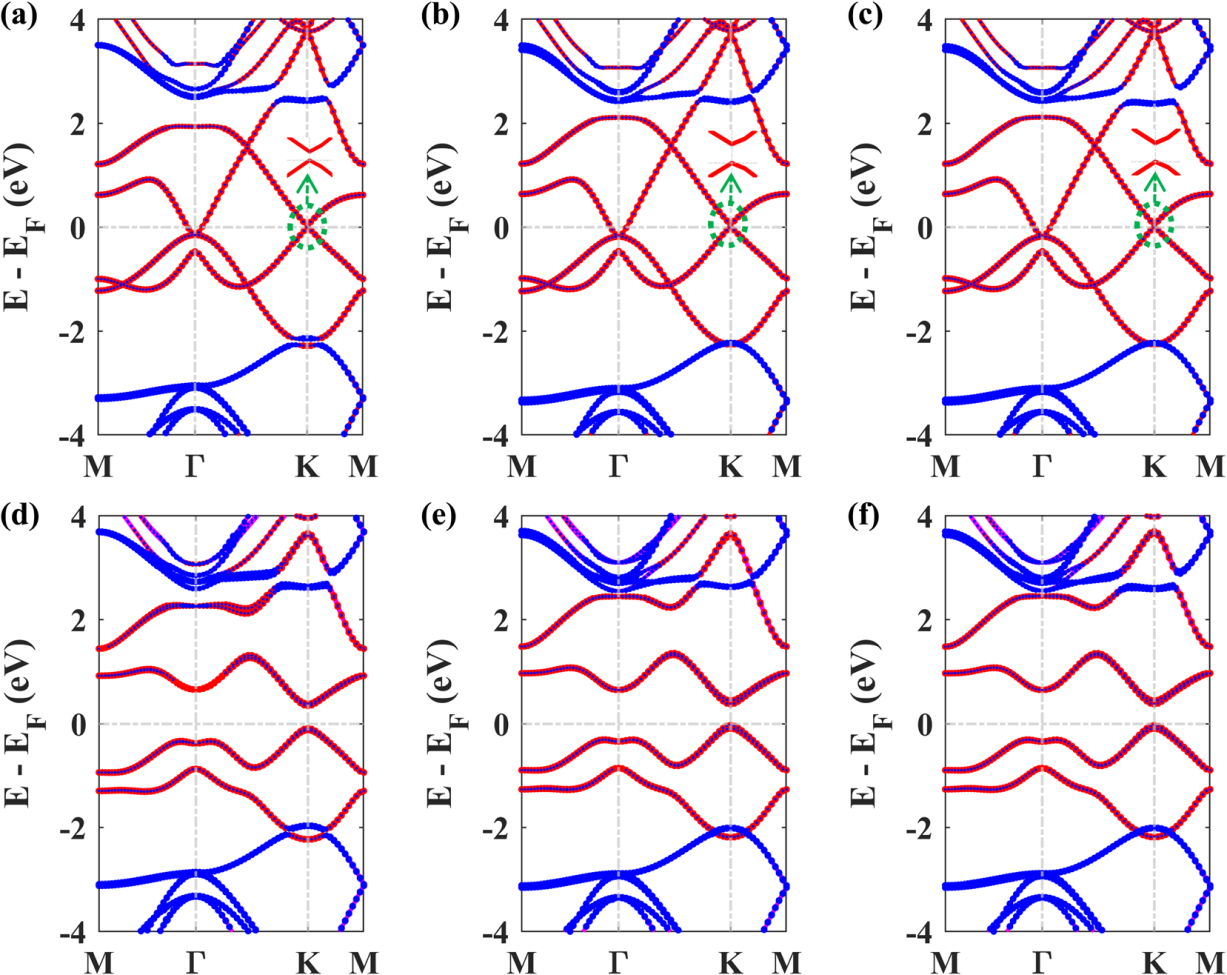
Band structure of the plumbene/hBN heterostructure: (a) structure I, (b) structure II, and (c) structure III in the absence of SOC. Band structure of the plumbene/hBN heterostructure: (d) structure I, (e) structure II, (f) structure III in the presence of SOC (red denotes the contribution of Pb, blue denotes the total contribution of B and N atoms).

Next, we studied the electronic band structures of the three stacking patterns. Fig. 4(a)–(c) display the band structures of the three configurations in the absence of SOC. All three band structures closely resemble each other, indicating that any of these three stacking patterns will generate similar electronic properties. When SOC is not considered, the Dirac cones are preserved, and bandgaps of 35.4 meV, 66.74 meV, and 65.2 meV are opened at the *K* point in structures I, II, and III, respectively, similar to the case of stanene/hBN.^5^

Fig. 4(d)–(f) display the band structures of the three structures in the presence of SOC. These three band structures are almost similar. From these atom-projected band structures, it is observed that the bands near the Fermi energy level (from −2 eV to 2 eV) are populated mainly by Pb atoms belonging to the plumbene layer, and the rest of the bands are primarily populated by B and N atoms belonging to the hBN layer. One important and interesting observation is that the VBM has been shifted to the *K* point, which was located near the *Γ* point in the pristine plumbene monolayer. Thus, using hBN as a substrate for the plumbene monolayer has resulted in the transition of indirect bandgap plumbene to a direct bandgap plumbene/hBN heterostructure.

Structures I, II, and III have direct bandgaps of 0.432 eV, 0.399 eV, and 0.402 eV, respectively, which are all greater than the indirect bandgap (0.34 eV) of pristine plumbene. Structures I, II, and III have electron effective masses of 0.2153*m*_o_, 0.2008*m*_o_, and 0.2017*m*_o_, and hole effective masses of 0.2101*m*_o_, 0.1962*m*_o_, and 0.1971*m*_o_ in that order. This preferable combination of low effective mass and significant direct bandgap makes the plumbene/hBN heterostructure a potential candidate for semiconductor and optoelectronic devices. As the band structures of the three stacking patterns look similar and because structure II was found to possess the lowest binding energy, we have considered structure II for the rest of the study. Since the PBE functional is known to underestimate the bandgap, we have also calculated the bandgap for structure II using the HSE06 functional. We employed a gamma centered *k* point grid size of 12 × 12 × 1 for sampling the BZ, and chose a 6 × 6 × 1 *q* mesh size for *q* sampling of the Fock operator. The calculated HSE bandgap including SOC is 0.694 eV.

**Fig. 5:**
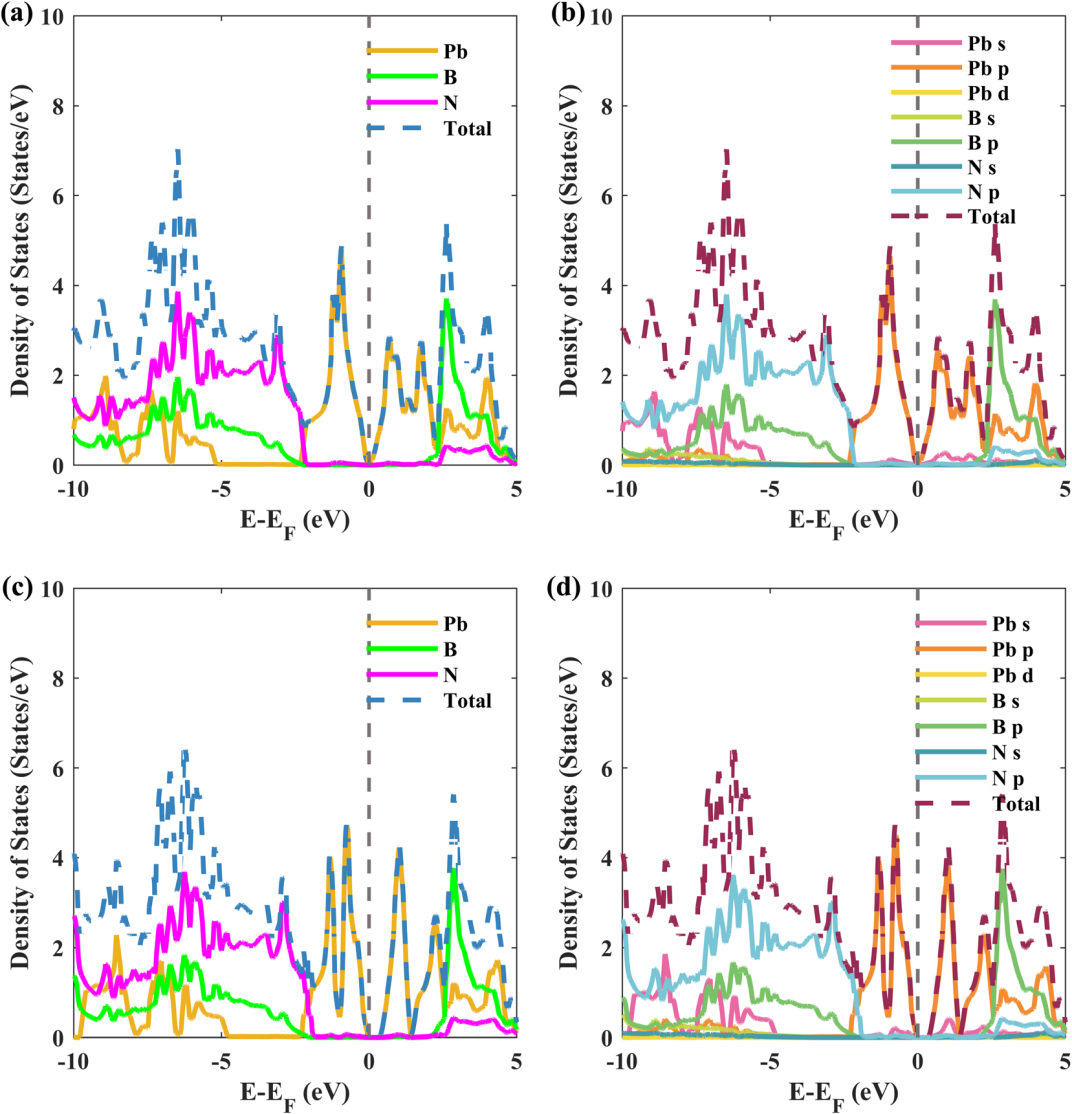
Density of states *versus* energy for structure II configuration of the Pb/hBN heterostructure. (a) Contributions from Pb, B, and N atoms (without SOC). (b) Contributions from each orbital of the atoms (without SOC). (c) Contributions from Pb, B, and N atoms (with SOC). (d) Contributions from each orbital of the atoms (with SOC).

To understand the interlayer interactions between the plumbene and hBN layers, the total and projected density of states (PDOS) for structure II of the Pb/hBN heterobilayer are calculated and plotted in Fig. 5. Fig. 5(a) and (b) illustrate the atom-resolved and orbital-resolved partial density of states (PDOS), respectively, without SOC. Fig. 5(c) and (d) show the corresponding PDOS with SOC. As can be seen from Fig. 5(a) and (c), plumbene predominately contributes to the conduction band (0 to 2.4 eV) and valence band (−2.4 to 0 eV) peaks of the PDOS. In addition, orbitals originating from B and N atoms do not contribute near the Fermi level. This indicates that the interactions between the two layers are trivial near the Fermi level. Therefore, it is expected that electronic carriers will only travel through the plumbene layer, leaving the hBN layer as a suitable substrate. Previous studies had also reported the preservation of bands of group IV monolayers near the Fermi level when the monolayers were stacked on the hBN layer.^21,36–38^ The p orbital of Pb atoms dominates the valence and conduction bands, which is evident from the orbital-resolved PDOS in Fig. 5(b) and (d). The states near the Fermi level originate from the unhybridized *p*_*z*_ orbital of Pb, implying that they are dominated mainly by π and π* orbitals of Pb atoms from the plumbene monolayer. When SOC is included, Fig. 5(c) and (d) reveal that the density of states is zero for a tiny area of energy from 0 eV to 0.346 eV with respect to the Fermi energy level, indicating the existence of a bandgap, as previously predicted from the band structures.

**Fig. 6:**
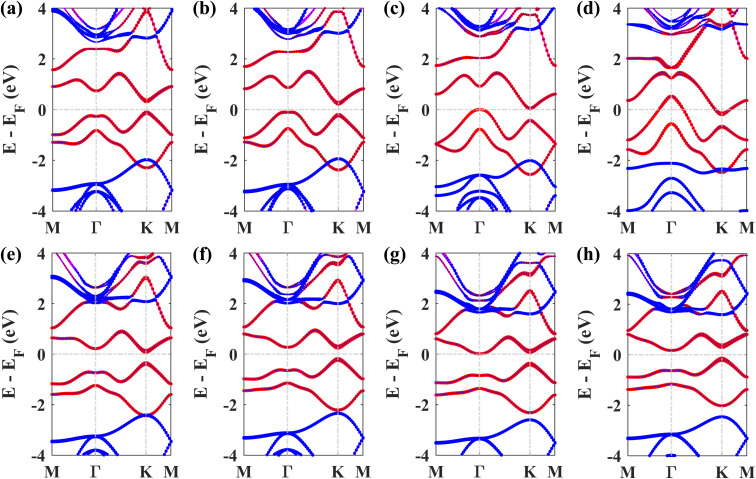
Band structures of the Pb/hBN heterobilayer in the presence of SOC with applied biaxial strain. (a) *ε* = −2%, (b) *ε* = −4%, (c) *ε* = −6%, (d) *ε* = −8% strain and (e) *ε* = 2%, (f) *ε* = 4%, (g) *ε* = 6%, (h) *ε* = 8% strain.

**Fig. 7:**
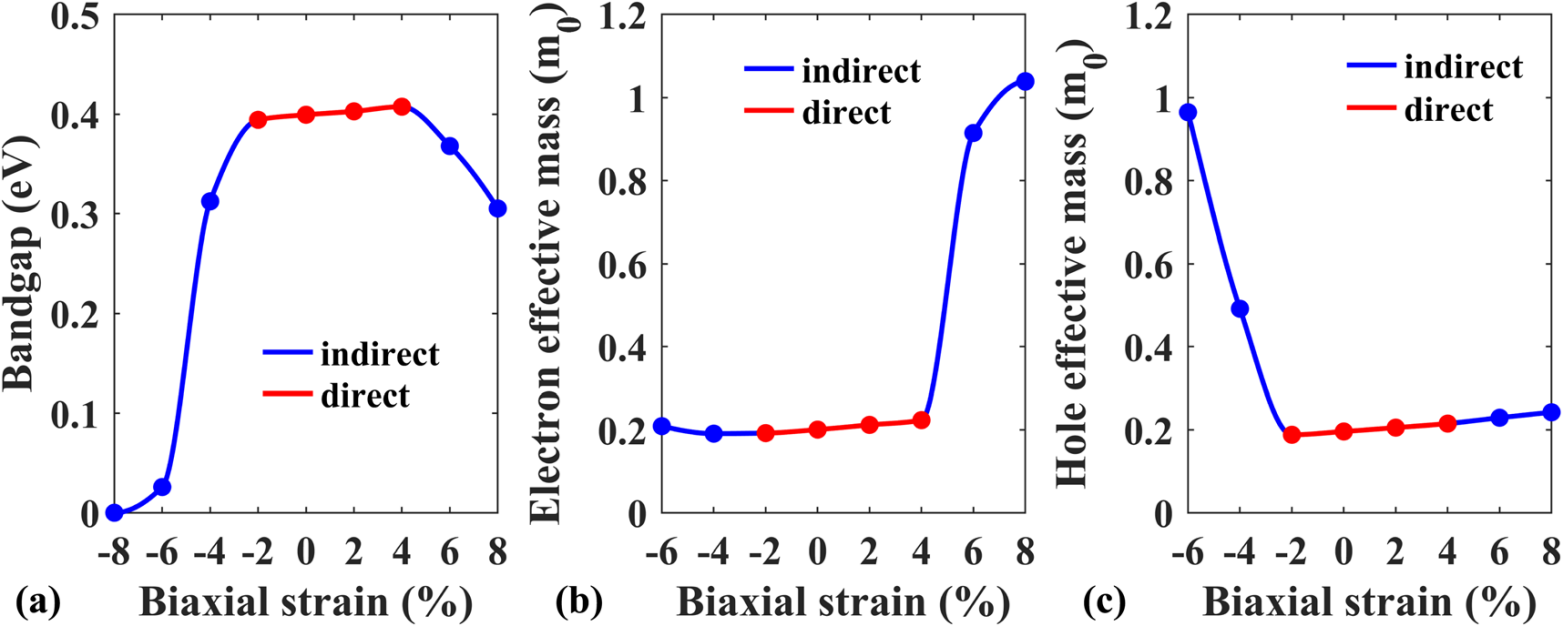
(a) Energy band gap, (b) electron effective mass, and (c) hole effective mass variation of the Pb/hBN heterostructure with biaxial strain.

Next, the effect of external biaxial strain on the electronic properties of the Pb/hBN heterostructure has been investigated. Fig. 6(a)–(d) represent the band structures of the Pb/hBN heterostructure with SOC for biaxial compressive strain ranging from −2% to −8%. The band structures demonstrate that when compressive strain is applied, the bandgap remains direct at −2% strain. At −4% strain, the bandgap becomes indirect. Here, the CBM lies at the same point as the unstrained structure. However, the VBM changes from *K* point to *Γ* point, resulting in a transition from direct to indirect bandgap. As the strain is increased, this indirect bandgap begins to diminish. Eventually, both the CBM and VBM cross the Fermi level at −8% strain, transforming the behavior of the heterostructure from semiconducting to metallic. Fig. 6(e)–(h) exhibit the band structures of the Pb/hBN heterostructure with biaxial tensile strain. At 2% tensile strain, it is observed that the bandgap is direct, and it remains so at 4% tensile strain as well. At 6% tensile strain, the bandgap becomes indirect. Here the VBM remains at *K* point, but the CBM shifts from *K* point to *Γ* point, causing a direct-to-indirect transition. As tensile strain is applied further, this indirect bandgap reduces with strain. This variation of the bandgap with biaxial strain is plotted in Fig. 7(a).

Fig. 7(b) depicts how electron effective mass can be modified with external biaxial strain. Applied tensile strain of up to 4% increases electron effective mass slightly. At 6% strain, the CBM is located at *Γ* point instead of *K* point, indicating a valley switching of electrons. As the curvature of the conduction band at *Γ* point is substantially less steep, the electron effective mass sharply rises beyond this point. On the other hand, no valley switching occurs when a compressive strain is applied, and the electron effective mass remains almost unchanged under compressive strain. Fig. 7(c) shows the variation of hole effective mass with applied biaxial strain. A slight reduction in the hole effective mass is noticed up to −2% strain. Beyond this strain, the hole effective mass sharply rises, which can be attributed to the shift of VBM from *K* point to *Γ* point. On the other hand, hole effective mass slightly increases with applied biaxial tensile strain.

**Fig. 8:**
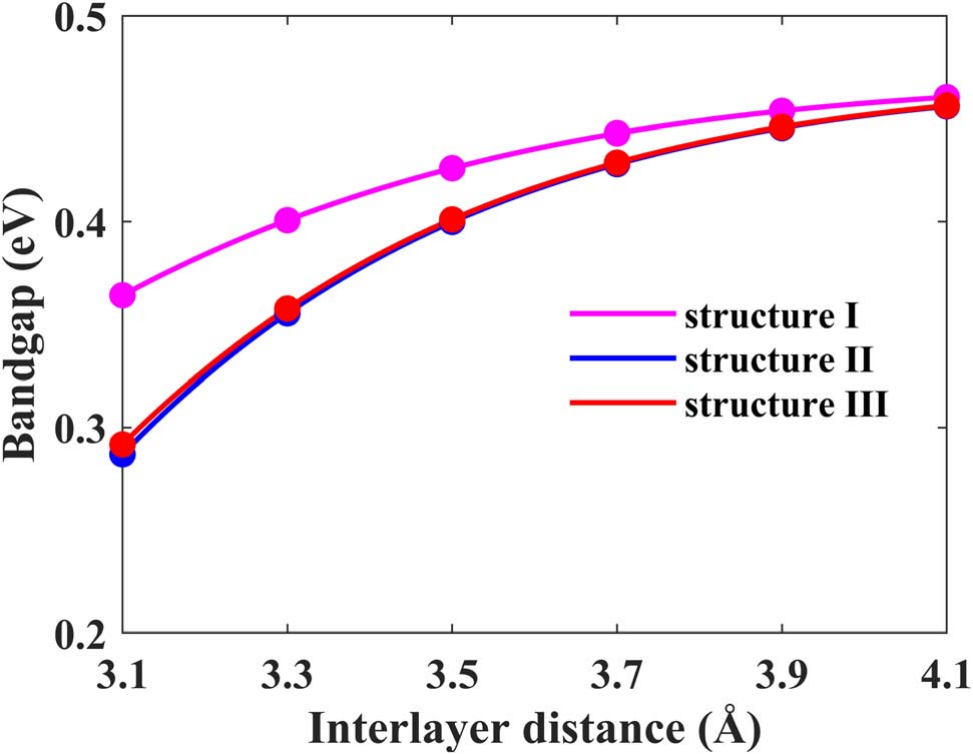
Energy band gap variation of the Pb/hBN heterostructure with interlayer distance for three different stacking patterns.

Another possible way of tailoring the electronic properties is by varying the interlayer distance between the monolayers. In this section, we focus on fine-tuning the bandgap of the Pb/hBN heterostructure using this approach. Firstly, the energy bandgaps for all three proposed stacking patterns have been calculated by changing the interlayer distance between the plumbene and hBN layers, as shown in Fig. 8. Above the optimal interlayer distance, the energy bandgap increases with an increase in the interlayer distance. On the other hand, the bandgap decreases with a decrease in interlayer distance as the interaction between the hBN and plumbene layers grows. This type of change in bandgap with interlayer distance has previously been reported in MoSSe bilayers.^39^ Interlayer interactions related to charge redistribution in the space separating the two layers contribute to this behavior. This further confirms the existence of both electrostatic and vdW interactions at the interface.

**Fig. 9:**
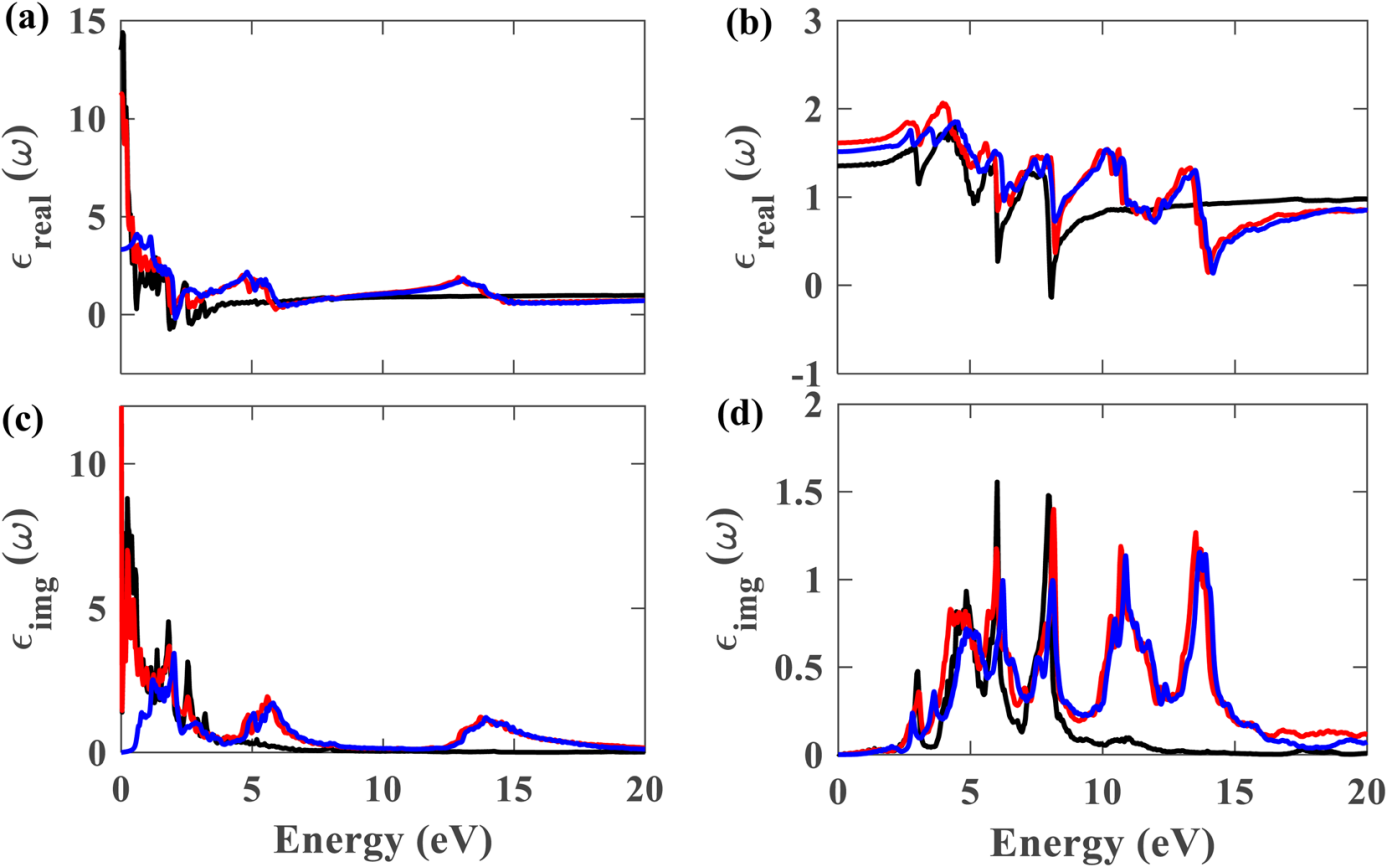
Real part of the dielectric function for (a) ∥ and (b) ⊥ polarization of light. Imaginary part of the dielectric function for (c) ∥ and (d) ⊥ polarization (black represents plumbene without SOC, red represents Pb/hBN without SOC, blue represents Pb/hBN with SOC + HSE).

From this point onwards, a study on the optical properties of the proposed heterostructure is presented. For benchmarking purposes, we calculated the complex dielectric function of graphene using QE. The shapes of the obtained dielectric function graphs closely match the corresponding reported graphs in the literature,^40^ indicating the reliability of our simulation methods. To evaluate the possibility of using the Pb/hBN heterostructure in photoelectronic devices, studying its optical properties is crucial. Fig. 9(a)–(d) display the complex dielectric function for the plumbene monolayer and Pb/hBN bilayer. Fig. 9(a) and (b) exhibit the real part for light polarized parallel and perpendicular to the plane of the materials, whereas Fig. 9(c) and (d) display the imaginary part for parallel and perpendicular polarization directions, respectively. The real part of the dielectric function corresponds to the dispersive effect, *i.e.*, the stored energy within the medium. On the other hand, the imaginary part of the dielectric function is related to the energy absorption within the medium. The static dielectric constant (*ε*_0_) is the dielectric constant indicating material behavior in low-frequency or constant electric fields. The values of *ε*_0_ are 3.3, 11.2, and 13.5 for the heterostructure considering SOC + HSE, without SOC, and the monolayer without SOC, respectively, for ∥ polarization of light. On the other hand, the corresponding values are 1.52, 1.62, and 1.36 for the ⊥ polarization. The higher *ε*_0_ of the Pb/hBN heterostructure along ⊥ polarization indicates its higher electromagnetic energy storage capacity than that of pristine plumbene along this direction. The oscillatory behavior of *ε*_real_ for the pristine plumbene monolayer stretches up to ∼8 eV, whereas in the Pb/hBN heterostructure, it stretches up to 15 eV along the ∥ direction. There are sharp peaks in the imaginary part of the dielectric function (*ε*_img_) below 4 eV for ∥ polarization of light for both pristine plumbene and the heterostructure (Fig. 9(c)). These peaks represent interband transitions in the visible and infrared regions. The imaginary part of the heterostructure has more peaks than pristine plumbene for both parallel and perpendicular polarization directions.

**Fig. 10:**
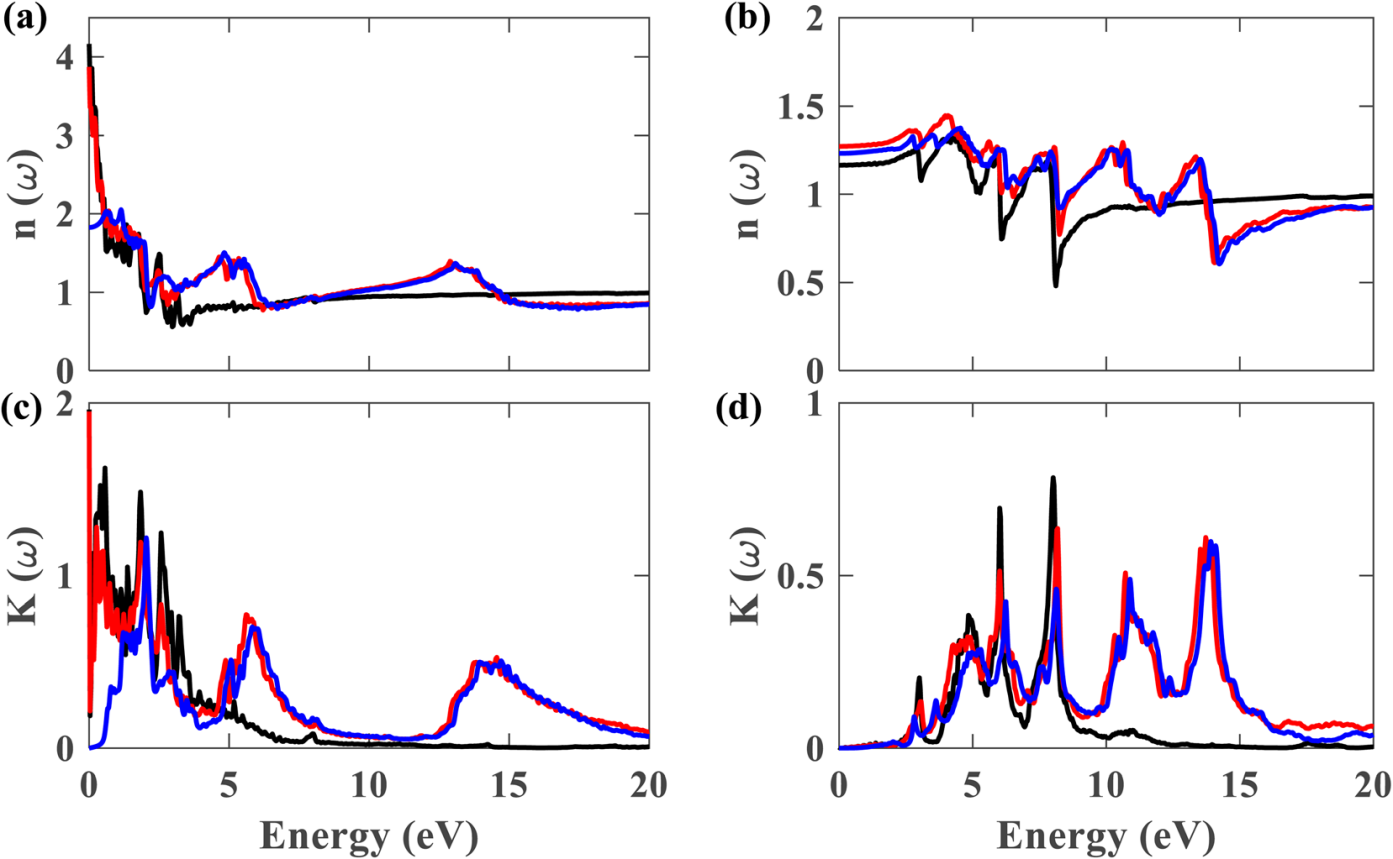
Real part of the refractive index for (a) ∥ and (b) ⊥ polarization of light. Imaginary part of the refractive index for (c) ∥ and (d) ⊥ polarization (black represents plumbene without SOC, red represents Pb/hBN without SOC, blue represents Pb/hBN with SOC + HSE).

Fig. 10(a)–(d) display the complex refractive index for the plumbene monolayer and Pb/hBN bilayer. Fig. 10(a) and (b) exhibit the real part of the complex refractive index for parallel and perpendicular polarization of light, respectively. The refractive index of a material is a fundamental optical property used to calculate a variety of other important properties. A material is said to have birefringence if the velocity of light differs in different polarization directions. The difference between the extraordinary and ordinary refractive indices is used to calculate birefringence. The refractive index for ∥ and ⊥ polarization directions is anisotropic in case of both the plumbene monolayer and Pb/hBN heterobilayer. Therefore, both of these materials show birefringence. Plumbene is isotropic above ∼15 eV, and plumbene/hBN becomes isotropic near ∼19 eV. The static refractive indices of the heterobilayer and plumbene are 3.87 and 4.17, respectively, for the ∥ direction, while they are 1.27 and 1.16 for the ⊥ direction in the absence of SOC. In the presence of SOC and HSE, the heterostructure has a refractive index of 1.82 along parallel and 1.23 along perpendicular polarization of light. A medium with a higher refractive index can bend light more; therefore, these materials can be of great interest in optoelectronic applications such as lenses. Fig. 10(c) and (d) exhibit the imaginary part of the complex refractive index or extinction coefficient for ∥ and ⊥ polarization directions, respectively. The extinction coefficient of the heterostructure demonstrates additional peaks to that of the plumbene monolayer in the UV region for both polarization directions.

**Fig. 11:**
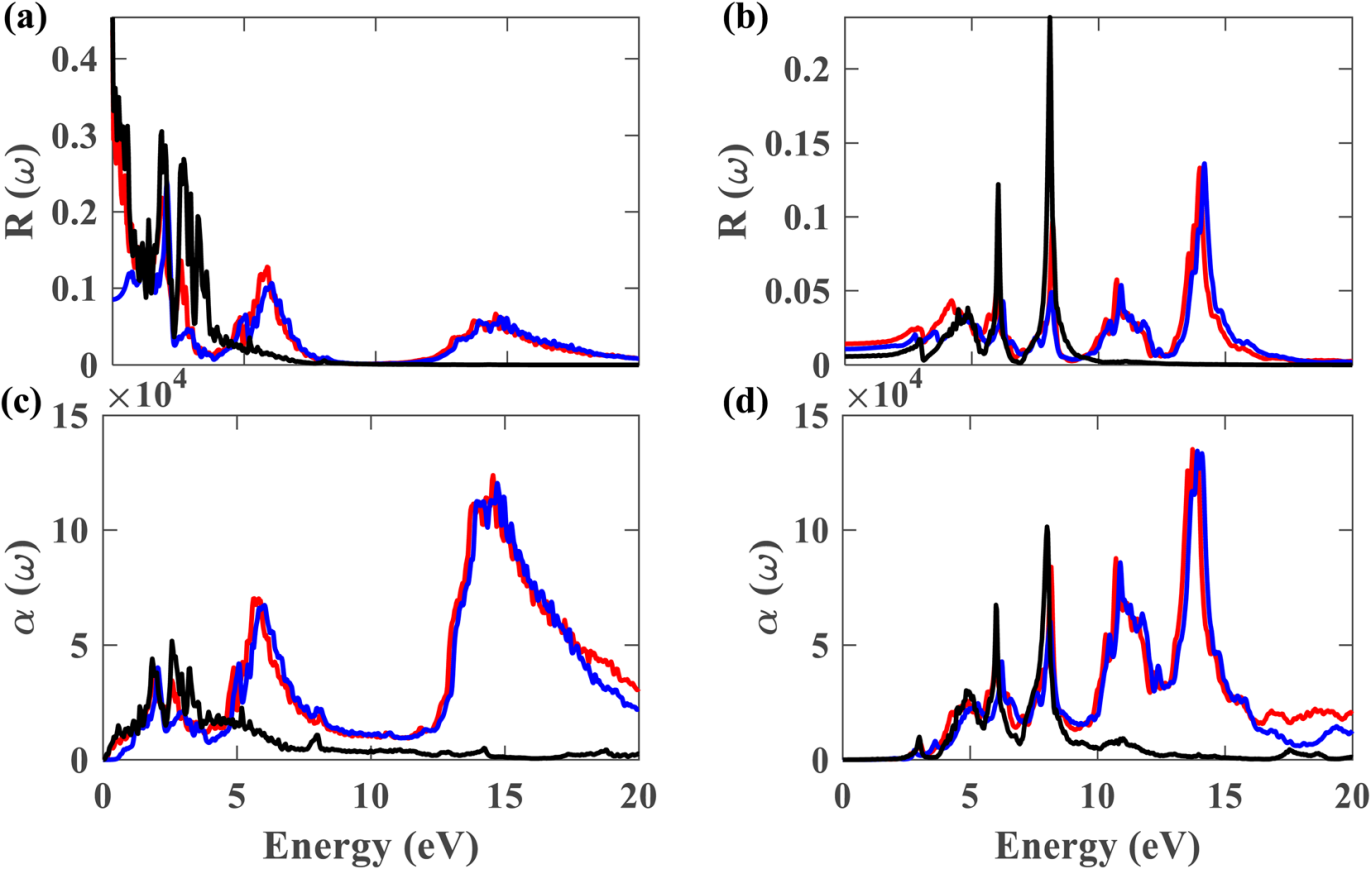
Reflectance for (a) ∥ and (b) ⊥ polarization of light. Absorption coefficient for (c) ∥ and (d) ⊥ polarization (black represents plumbene without SOC, red represents Pb/hBN without SOC, blue represents Pb/hBN with SOC + HSE).

Fig. 11(a) and (b) depict the reflectivity for ∥ and ⊥ polarization directions, respectively, for pristine plumbene and plumbene/hBN heterostructure. For the plumbene monolayer, reflectivity for the ∥ polarization direction is between 0.1 and 0.3 reaching up to ∼4 eV. The reflectivity values of the heterostructure lie in the same range up to ∼3 eV. The heterostructure exhibits minor reflectivity peaks around 6 eV and 14.6 eV for the ∥ direction. The bilayer shows additional peaks at ∼10.7 eV and ∼14 eV along the ⊥ polarization direction compared to the monolayer; the other reflectance peaks are observed below 10 eV. For the ⊥ direction, both materials show low reflectance (below 0.05) in the visible region, whereas for the ∥ direction, the reflectance value is below 0.1 in the UV region. A lower reflectance value denotes a higher probability of light transmission through the material, making it transparent to light belonging to those portions of the spectrum.

Fig. 11(c) illustrates the absorption coefficient of the plumbene monolayer and Pb/hBN bilayer for parallel polarization. Plumbene has one prominent optical absorption band from 0.5 to 6 eV. These values encompass the visible, near-UV, and mid-UV portions of the electromagnetic spectrum. In contrast, the heterostructure contains three significant absorption bands from 1.4 to 3.2 eV, 4.5 to 7.7 eV, and 12.6 to 20 eV covering visible, mid, and far UV domains. Fig. 11(d) depicts the absorption coefficient for the plumbene monolayer and Pb/hBN bilayer for perpendicular polarization of light. Plumbene has two high optical absorption zones: 4.2 to 6.6 eV and 7 to 8.6 eV. These values encompass the middle and far ultraviolet spectrum.

In contrast, the heterostructure contains four significant absorption zones: 4.1–6.8 eV, 7.3–8.8 eV, 9.9–12.2 eV, and 12.9–15 eV, spanning the mid, far, and extreme UV spectrum. In the SOC + HSE approach, the heterostructure exhibits four major peaks at 6.24, 8.11, 10.89, and 13.9 eV along the ⊥ polarization direction. Both materials have absorption coefficients of the order of 10^4^ in the visible region, indicating that both structures are potential candidates for optoelectronic devices that work in the visible range, such as solar cells. The interactions due to the heterostructure give rise to an enhanced absorption coefficient in the UV region which encourages the application of the Pb/hBN heterostructure in UV photodetectors as well. Ultraviolet (UV) photodetectors can find applications in advanced communications, air filtration, ozone monitoring, leak identification, and flame detection.^41^

**Fig. 12:**
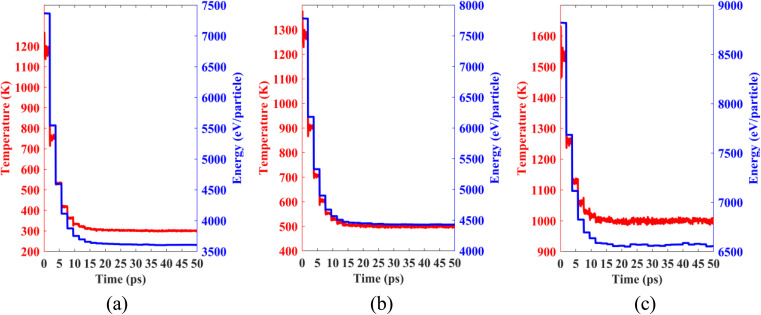
Temperature and total energy fluctuations of MD simulations carried out at (a) 300 K, (b) 500 K and (c) 1000 K.

Lastly, we performed classical molecular dynamics simulations at constant temperatures of 300 K, 500 K, and 1000 K to study the thermal stability of the heterobilayer. Temperature *vs.* time and total energy *vs.* time graphs are plotted in Fig. 12. The plots show that temperature and total energy converge with minor fluctuations at equilibrium. No major structural reconstruction takes place at any of the three temperatures. These results indicate the thermal stability of the heterostructure at and above room temperature.

## Conclusion

In this work, a thorough investigation of the structural, electronic, and optical characteristics of the Pb/hBN heterobilayer has been conducted using first-principles calculations. Three different stacking configurations have been studied, all of which are electronically stable with negative binding energies. The difference in binding energies is very small for different configurations, which establishes the insensitivity of the heterostructure to different stacking patterns. The band structures exhibit direct bandgaps of 0.432 eV, 0.399 eV, and 0.402 eV at *K* point, which are higher than the indirect bandgap of pristine plumbene (0.34 eV). The projected density of states shows the preservation of electronic properties of the plumbene monolayer, proving the feasibility of hBN as an ideal substrate for the experimental realization of plumbene. Additionally, the Pb/hBN heterostructure has potential for improved charge carrier transport based on the estimated low electron and hole effective mass. Our findings further show that the bandgap can be effectively controlled while maintaining stability by varying the interlayer distance between the plumbene and hBN monolayers and applying external biaxial strain. At 8% biaxial compressive strain, a semiconductor to metal transition occurs. Such exciting and tunable electronic properties of the Pb/hBN heterobilayer can highly motivate Pb-based electronic device applications. The optical properties reveal that the heterostructure shows anisotropic behavior in different polarization directions (along ⊥ and ∥ directions). Also, the composite structure helps enhance the absorption properties of pristine plumbene. The heterostructure has a very high absorption coefficient (in the order of 10^4^ cm^−1^) in both visible and UV regions, indicating its potential application in solar cells and UV photodetectors. Finally, the excellent findings in this study can inspire the emergence of Pb-based novel nanoelectronic and optoelectronic devices.

## Conflicts of interest

There are no conflicts of interest to declare.

## Supplementary Material
